# Development of a Biosensor-Based Rapid Urine Test for Detection of Urogenital Schistosomiasis

**DOI:** 10.1371/journal.pntd.0003845

**Published:** 2015-07-02

**Authors:** Kathleen E. Mach, Ruchika Mohan, Shailja Patel, Pak Kin Wong, Michael Hsieh, Joseph C. Liao

**Affiliations:** 1 Department of Urology, Stanford University School of Medicine, Stanford, California, United States of America; 2 Department of Aerospace and Mechanical Engineering, The University of Arizona, Tucson, Arizona, United States of America; 3 Veterans Affairs Palo Alto Health Care System, Palo Alto, California, United States of America; Centers for Disease Control and Prevention, UNITED STATES

## Introduction

Schistosomiasis affects up to 300 million people in regions of South America, Southeast Asia, the Middle East, Mediterranean Europe, and sub-Saharan Africa. Chronic consequences of schistosomiasis include anemia, physical and cognitive retardation in children, and organ failure. With predilection for the genitourinary tract, *Schistosoma haematobium* increases the risk of bladder cancer and HIV infection in women. Microscopic identification and enumeration of parasite eggs in urine (*S*. *haematobium*) or stool (*Schistosoma mansoni* and *Schistosoma japonicum*) has remained the standard for schistosomiasis diagnosis [1]. Limitations of microscopy include the need for skilled personnel in the field with microscopy equipment and its time-consuming nature. Particularly in infrastructure-limited regions, point-of-care (POC) molecular diagnostics hold the potential to transform the management of infectious diseases such as schistosomiasis that carry significant long-term morbidity if left undiagnosed. POC diagnosis could allow selective drug administration to individuals with confirmed infection rather than entire schools or communities, and an integrated diagnosis–single dose therapy approach could reduce costs of drug administration campaigns and minimize treatment-associated adverse effects.

**Fig 1:**
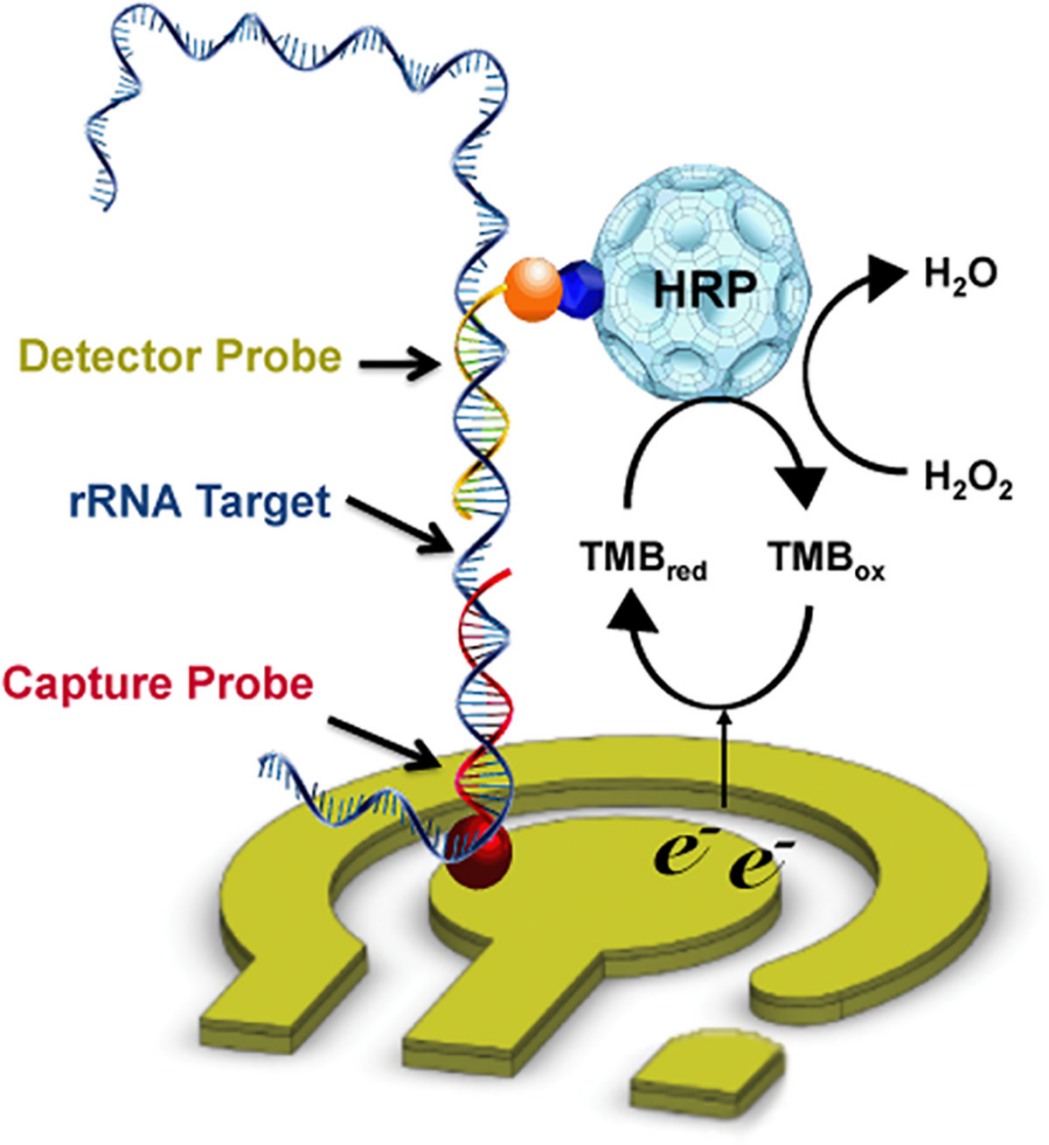
Biosensor-based molecular detection of urinary pathogen. The biosensor is composed of three planar gold electrodes (working, auxiliary, and reference). For the biosensor assay, capture probes are bound to the surface of the working electrode via a thiol linkage. Cells in the sample are lysed and mixed with a buffered solution of detector probe, then applied to the sensor surface. If the target rRNA is present, a hybridization complex of target, capture, and detector probes forms. This complex is detected by binding of horseradish peroxidase (HRP)-conjugated antifluorescein binding to a fluorescein tag on the detector probe and addition of tetramethylbenzidine (TMB) substrate. The electron transport mediated by the HRP is measured amperometerically, and the signal is proportional to the quantity of the target.

Electrochemical biosensors are well suited for molecular diagnostics because of their high sensitivity, low cost, ease of integration into POC devices, and portability of the reader instrumentation [2]. We have developed a strategy for rapid (one hour) molecular diagnosis of bacterial urinary tract infections using electrochemical biosensors [3,4]. Urinary cells are lysed and directly applied to an array of sensors functionalized with oligonucleotide probes targeting the 16S ribosomal RNA (rRNA) of common uropathogens [3,4]. Formation of the sequence-specific hybridization complex between the pathogen rRNA and the labeled capture and detector probe pairs is detected by an enzyme tag that mediates an amperometric signal output (Fig 1).

In this work, we demonstrate the use of our biosensor-based platform for detection of *S*. *haematobium* in urine. We developed capture and detector probes targeting *S*. *haematobium* rRNA and integrated them into our established molecular diagnostics strategy. After determining an efficient egg lysis strategy, we demonstrated direct electrochemical detection of *S*. *haematobium* eggs spiked in human urine.

## Development and Validation of a Urine Test for *S*. *haematobium*

### Probe design and analysis

Using Geneious 7.0.3 software, available rRNA sequences from GenBank, and published PCR primers [5–7], we identified five candidate probe pairs homologous to *S*. *haematobium* 18S or 28S rRNA. Similarity searches of the probe sequences using BLAST (http://blast.ncbi.nlm.nih.gov/Blast.cgi) indicate they likely bind other schistosome species such as *S*. *mansoni* and *S*. *japonicum* because of the high degree of homology in their rRNA genes, but no significant homology outside the genus *Schistosoma* was identified. Given only *S*. *haematobium* eggs are generally found in urine, we determined that the sequences would confer specificity for *S*. *haematobium* when testing human urine samples (Table 1).

**Table 1:** Sequences of capture and detector probe pairs tested for detection of *S. hematobium* ^a^.

Probe name	Sequence (5'-3')^b^
18SH323	
Capture	AAGTTATCCAGAGTCATCACAG
Detector	AATGTARCAGGCACAGCCGAAG
18SH758	
Capture	TCCTGATCGTAACCAAAACCGT
Detector	GAACAAGCAGCGGCATGCGACC
28SH495	
Capture	CGACTCCAAGGGTAGCTCAGAR
Detector	CARAACTGTCACACTTGATCTC
28SH521	
Capture	ACTTTGGGCTGCATTCACAAAC
Detector	AACCCGACTCCAAGGGTAGCTC
28SH693	
Capture	CACACTCATCAGCTGAATTC
Detector	CCAGAGCTTGCAGTTCAACTC
PC1	
Capture	ACTACCTACAATAAAAAACTCC
Detector	AAAAATAAGTCGCACAAACATC
NC1	
Capture	TCCCACACCCCACGATACAAAA

^*a*^ The capture probes were modified with 5’ thiol, and detector probes were modified with 3’ fluorescein.

^*b*^ The degenerate base “R” represents either A or G.

**Fig 2:**
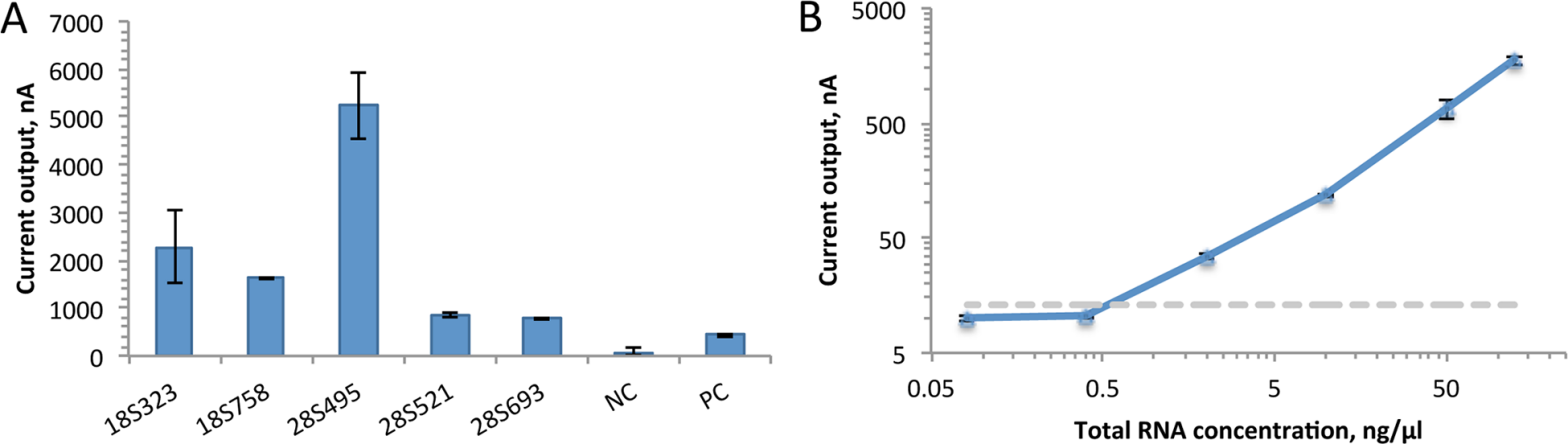
*S*. *haematobium* probe testing. **A**. Sensors were functionalized with the different candidate capture probes for *S*. *haematobium* detection or positive and negative control probes. A 25 ng/μl solution of total RNA with the appropriate matching detector probe was applied to sensors with candidate probes, a 0.5 nM solution of synthetic target with matching detector probe was applied to sensors for positive control, and a hybridization solution with no target was applied to negative control sensors. The highest signal of 5237 nA for the 28S495 probe set indicated this probe would give the best sensitivity for *S*. *haematobium* detection. **B.** To determine the limit of detection for the 28S495 probe set, all sensors on the chip were functionalized with the 28S495 capture probe. Serial dilutions of *S*. *haematobium* total RNA were applied to the sensors for assay.

We tested the probe pairs in our electrochemical biosensor assay with *S*. *haematobium* RNA prepared from adult worms by the Schistosome Research Reagent Resource Center (distributed by BEI Resources). Standard chip arrays consisting of 16 electrochemical sensors were purchased from GeneFluidics (Irwindale, USA). The individual sensors were functionalized with thiolated capture probes and positive and negative control sequences as previously described [8], and the biosensor assay was conducted as previously reported [4]. Hybridization to *S*. *haematobium* 18S rRNA (18S323, 18S758C) and 28S rRNA (28S495, 28S521, and 28S693) probes was tested at 50°C. The 28S495 probe pair yielded the highest signal in the biosensor assay (Fig 2A) and was chosen for further assay development.

### Ethics statement

All animal procedures were conducted according Administrative Panel on Laboratory Animal Care (APLAC) approved protocol #22502 and Veterinary Service Center institutional guidelines of Stanford University (Animal Welfare Assurance A3213-01 and USDA License 93-4R-00). For *S*. *haematobium* egg isolation, each hamster was anesthetized with an intraperitoneal injection of 500 μL of a 0.504 mg (100.8 units/mL) solution of heparin sodium (Sigma-Aldrich) and 26 mg/mL sodium pentobarbital. Once under deep general anesthesia (including no withdrawal responses to paw pinch), each hamster underwent a combined thoracotomy and laparotomy for euthanasia and to expose the abdominal viscera. The liver and intestines of each hamster were harvested, and *S*. *haematobium* eggs were isolated.

### Analytical sensitivity of the electrochemical biosensor assay with *S*. *haematobium* egg RNA

To verify the analytical sensitivity of the probe pairs, we isolated total RNA directly from *S*. *haematobium* eggs. Tissues from *S*. *haematobium*-infected Lakeview Golden (LVG) hamsters obtained from the Schistosomiasis Research Reagent Resource Center (Biomedical Research Institute, Rockville, Maryland) were harvested for egg isolation at approximately 18 weeks postinfection [9]. After harvesting, *S*. *haematobium* eggs were preserved in RNA Later (Ambion, Life Technologies). Total RNA from approximately 8,000 eggs was extracted using Trizol Reagent (Ambion, Life Technologies, USA) according to manufacturers' instructions. To determine the limit of detection (LOD) of the biosensor assay for *S*. *haematobium* egg RNA, serial dilutions of the purified RNA were prepared and tested. Using the 28S495 probe pair, as little as 0.53 ng/μl total egg RNA from *S*. *haematobium* egg rRNA was detected in the biosensor assay (Fig 2B).

### Direct detection of 28S rRNA from *S*. *haematobium* eggs

Next, we tested different egg lysing strategies to enable direct detection of *S*. *haematobium* rRNA in the crude lysate using the biosensor assay. Mechanical lysis offers high versatility and is amenable to integration in diagnostic devices [10]. We compared two different lysis strategies: glass bead agitation and sonication.

**Fig 3:**
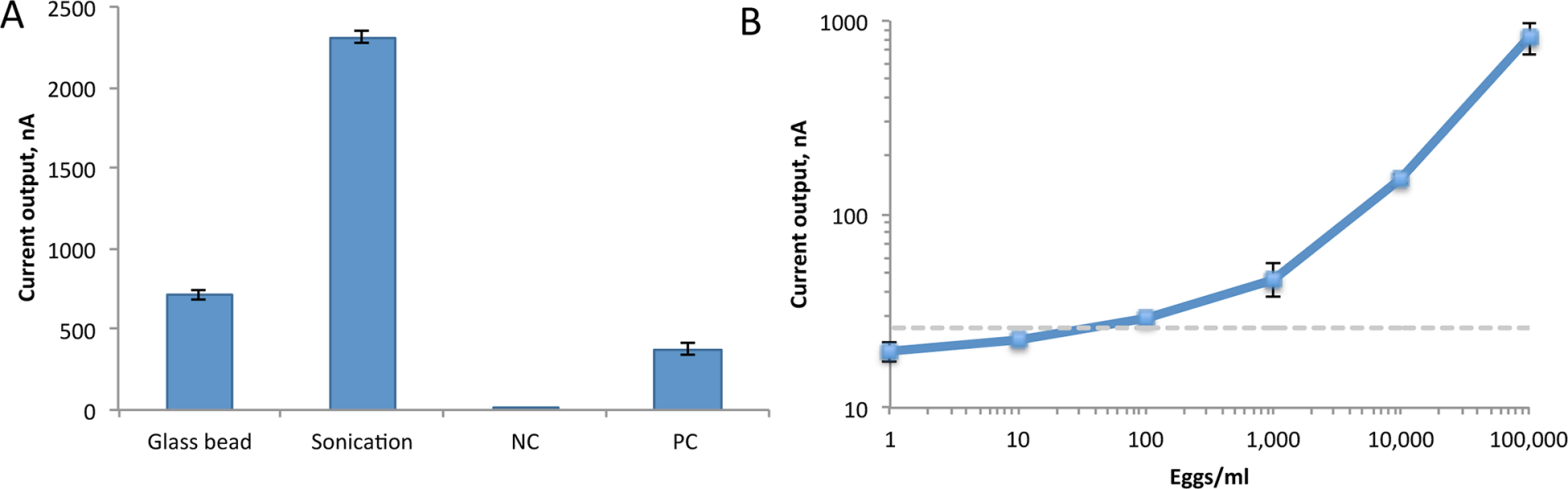
Detection of *S*. *haematobium* eggs. A. *S*. *haematobium* eggs spiked in human urine were lysed by agitation with glass beads or sonication and the crude lysate tested in the biosensor assay with the 28S495 probe set with positive and negative controls as described in Fig 2. The higher signal with sonication compared to glass beads indicates more efficient cell lysis. B. To determine the LOD for detection of *S*. *haematobium* eggs in urine in the biosensor assay, a sample of 10^6^ egg/ml was lysed by sonication and serially diluted in urine. Biosensor assay detection of *S*. *haematobium* eggs with the 28S495 probe set indicated an LOD of approximately 30 eggs/ml.

For each lysis protocol, approximately 10^6^ eggs were suspended in 500 μl lysis buffer [0.1% Triton X-100, 20 mM tris-HCl (pH 8.0), 2 mM EDTA (pH 8.0)] in a 1.5 ml tube. For glass bead agitation, 10–15 glass beads, (1 mm diameter, Sigma-Aldrich) were added to the tube containing eggs in lysis buffer then vigorously vortexed for 2 min. The beads were allowed to settle and the lysate removed for assay. For sonication, the eggs suspended in lysis buffer were subjected to two 10-sec pulses with a probe sonicator (Sonic Dismembrator Model 100, Fisher Scientific) at output power of 4–5 RMS with 10 sec on ice between sonication to prevent excess RNA damage. To avoid cross-contamination, the sonicator probe was cleaned with isopropanol between uses. The crude lysate was then used in a biosensor assay. Starting with the same number of eggs for both lysis protocols, the biosensor signal was 3.3-fold higher after lysis by sonication compared to agitation with glass beads (Fig 3A). The data suggest that sonication yield more efficient liberation of rRNA from the schistosome eggs and was used for lysis in subsequent experiments.

### Detection of *S*. *haematobium* eggs in urine with electrokinetic-facilitated hybridization

Previously, we integrated alternating current (AC) electrokinetics to improve direct electrochemical detection of bacterial pathogens in urine [8,11]. By inducing bulk fluid motion and local heating, AC electrokinetics improved overall signal-to-noise of the biosensor assay. Further, implementation of electrokinetics will facilitate integration into a POC device as it obviates the need for an external incubator for hybridization. For schistosomal detection, we applied square wave AC potential across the working and auxiliary electrodes of the electrochemical sensors using a function generator (HP, 33210A) and a setting of 200 kHz, 7 Vpp for 15 minutes. We determined the LOD of *S*. *haematobium* eggs in human urine with probe pair 28S495 with electrokinetics-facilitated hybridization. Urine containing *S*. *haematobium* eggs was sonicated and serially diluted in the urine sample, and dilutions were tested in the biosensor assay. The LOD was approximately 30 eggs/ml urine in the biosensor assay. However, optimization of sensitivity, possibly through concentration of eggs in urine or biosensor probe modification, will be necessary for detection of light infections where as few as 2–3 eggs/ml may be present in urine [12].

## Conclusion

This study is an important step toward development of a POC device for rapid detection of *S*. *haematobium* eggs in urine (Box 1). We have implemented strategies that will aid in device integration, such as mechanical lysis and AC electrokinetics. For future development, we will integrate this core assay into a fully automated microfluidics cartridge, further optimize the detection sensitivity, and validate with clinical samples.

**Box 1. Advantages and Disadvantages of a Biosensor-Based Rapid Diagnostic Test for Detection of Urogenital Schistosomiasis**

Advantages

- Biosensor-based approach offers quantitative detection of parasite-derived nucleic acids, which is potentially less subjective to the current standard based on egg counting.
- Does not require shedding of eggs for detection of parasite nucleic acids in urine (particularly relevant for diagnosis of low-intensity infection).
- Time-to-result is on the scale of several minutes rather than hours.

Disadvantages

- Sensitivity for detecting low intensity infections likely still not optimal.
- Current version of biosensor-based diagnostic test is not POC.
- Test still requires trained users rather than being usable by lay persons.

### Sequence numbers

Biosensor probes based on GenBank sequences Z11976.1 and Z46521.
